# Granular Cell Tumor of the Breast: It is Time to Attach Importance to this Rare but Insidious Disease

**DOI:** 10.5334/jbr-btr.870

**Published:** 2015-12-30

**Authors:** Alessandro Fancellu, Alberto Porcu

**Affiliations:** 1 University of Sassari, Unit of General Surgery 2 - Dept. of Clinical and Experimental Medicine, V.le San Pietro, 43. Sassari 07100, Italy

Dear Editor,

We read with interest the article from Huyskens and Geniets [1] reporting on a case of granular cell tumor (GCT) of the breast in a 30-year-old woman. The case described herein recalls similar incidences observed at our institution. The first was in a 42-year-old woman where the clinico-radiological aspect closely resembled a carcinoma. Since fine needle aspiration cytology examination was inconclusive, surgical removal of the lesion allowed the correct diagnosis, thus avoiding inappropriate radical surgery not justified by the usual benign behavior of the neoplasm [2]. More recently, an additional case was seen in a 56-year-old woman (Figure 1). In this patient, the preoperative diagnosis of granular cell tumors (GCT) was established by core needle biopsy and the lesion was surgically removed.

**Figure 1:**
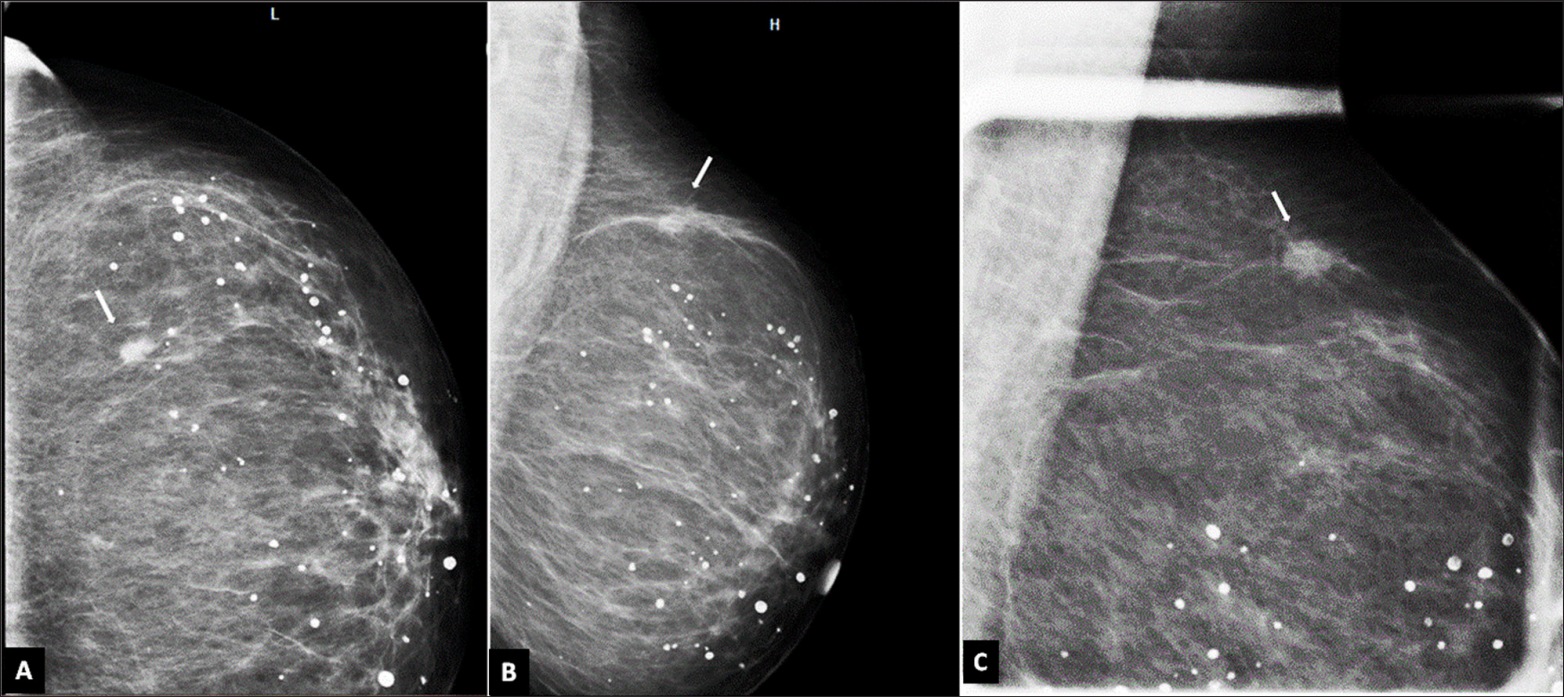
Mammography showing an irregular, speculated mass in the upper outer quadrant of the left breast (line). A, Craniocaudal projection; B and C, lateral projections.

Awareness of the existence of this pathological entity is the key to correct diagnosis by core needle biopsy, even in male patients with breast lumps [3]. GCT has also been described in coexistence with ductal adenocarcinoma of the breast. Diagnosis of GCT of the breast can also be reached using fine-needle aspiration cytology under ultrasound guidance in expert hands. We do agree with the authors’ approach in that they advocated surgical excision of the GCT. In fact, it should be emphasized that surgical excision is always warranted for those rare tumors, which *usually* have a benign behavior. This concept is reinforced when dealing with young women with biopsy proven GCT of the breast. Interestingly, several cases reported in literature were observed in patients in their 30s and 40s. Since the patient in the report by Huyskens and Geniets [1] refused surgical treatment, a strict clinical and imaging follow-up is mandatory. On the other hand, several authors reported on malignant forms of GCT of the breast with metastatic spread as well as locally advanced tumors [4]. Many of these patients would probably have had a better prognosis if they had undergone surgical excision of the tumor at the time of primary presentation. Furthermore, the risk of local recurrence cannot be underestimated. It is important to remove the tumor with adequate free margins, because local recurrences may pose diagnostic difficulties on imaging evaluation and may bring the risk of potentially disfiguring re-excisions.

When looking at the literature of GCT of the breast, several single-case descriptions with limited reviews can be found, reporting mostly on benign forms treated with local excision. However, solid conclusions from a comprehensive global review of all the reported cases are lacking. GCT of the breast is considered a rare usually benign, but sometimes insidious disease, which deserves further attention. A comprehensive and analytic review would help to better define imaging features, histological patterns, potential for malignant transformation and radiological-pathological correlation, all of which would be useful in order to propose appropriate management.

## Competing Interests

The authors declare that they have no competing interests.
